# The Sensory Perception Quotient (SPQ): development and validation of a new sensory questionnaire for adults with and without autism

**DOI:** 10.1186/2040-2392-5-29

**Published:** 2014-04-24

**Authors:** Teresa Tavassoli, Rosa A Hoekstra, Simon Baron-Cohen

**Affiliations:** 1 Department of Psychiatry, Autism Research Centre, Cambridge University, 18b Trumpington Road, Cambridge CB2 8AH, UK; 2 Seaver Autism Center, Icahn School of Medicine, 1428 Madison Avenue, 10129 New York, USA; 3 Department of Life Health and Chemical Sciences, The Open University, Milton Keynes MK7 6AA, UK; 4 Cambridgeshire and Peterborough NHS Foundation Trust, CLASS Clinic, Cambridge CB21 5EF, UK

**Keywords:** autism spectrum conditions, sensory questionnaire, sensory perception quotient

## Abstract

**Background:**

Questionnaire-based studies suggest atypical sensory perception in over 90% of individuals with autism spectrum conditions (ASC). Sensory questionnaire-based studies in ASC mainly record parental reports of their child’s sensory experience; less is known about sensory reactivity in adults with ASC. Given the DSM-5 criteria for ASC now include sensory reactivity, there is a need for an adult questionnaire investigating basic sensory functioning. We aimed to develop and validate the Sensory Perception Quotient (SPQ), which assesses basic sensory hyper- and hyposensitivity across all five modalities.

**Methods:**

A total of 359 adults with (n = 196) and without (n = 163) ASC were asked to fill in the SPQ, the Sensory Over-Responsivity Inventory (SensOR) and the Autism-Spectrum Quotient (AQ) online.

**Results:**

Adults with ASC reported more sensory hypersensitivity on the SPQ compared to controls (*P* < .001). SPQ scores were correlated with AQ scores both across groups (r = .-38) and within the ASC (r = -.18) and control groups (r = -.15). Principal component analyses conducted separately in both groups indicated that one factor comprising 35 items consistently assesses sensory hypersensitivity. The SPQ showed high internal consistency for both the total SPQ (Cronbach’s alpha = .92) and the reduced 35-item version (alpha = .93). The SPQ was significantly correlated with the SensOR across groups (r = -.46) and within the ASC (r = -.49) and control group (r = -.21).

**Conclusions:**

The SPQ shows good internal consistency and concurrent validity and differentiates between adults with and without ASC. Adults with ASC report more sensitivity to sensory stimuli on the SPQ. Finally, greater sensory sensitivity is associated with more autistic traits. The SPQ provides a new tool to measure individual differences on this dimension.

## Background

In addition to the classic diagnostic criteria (social and communication difficulties alongside unusually narrow interests and repetitive behaviour) [1] atypical sensory reactivity is now also recognized as being at the core of autism spectrum conditions (ASC) [2,3]. Under Symptom B the new autism criteria for DSM-5 include ‘Hyper- or hyporeactivity to sensory input or unusual interests in sensory aspects of the environment (for example, apparent indifference to pain/temperature, adverse response to specific sounds or textures, excessive smelling or touching of objects, visual fascination with lights or movement.’ [4]. The study of atypical sensory reactivity in individuals with autism is important given how common this is in ASC. Questionnaires are widely used to study sensory reactivity issues in children with and without ASC [5], in Sensory Processing Disorder [6] and other conditions [7]. Sensory questionnaire studies in ASC mainly record parental reports of their child’s sensory experience [5,8-16]. Although parent reports are an important tool, self-report is also crucial, as sensory experiences are by definition subjective. Only a few questionnaire studies have investigated sensory reactivity issues in adults with ASC [17,18].

The most widely used measure for adults with ASC is the Adolescent/Adult Sensory Profile (AASP), a 60-item self-report measure that finds differences in sensory processing in more than 90% of adults with ASC [17,19]. There have been several studies using the AASP in ASC; one of them found that older individuals with ASC are more similar to control groups than are younger individuals [20]. In addition, adults with schizophrenia and bipolar disorder also show differences on the AASP compared to controls [21]. The AASP is a useful tool because it can be used in clinical settings and describes the particular sensory problems of an individual by assessing specific sensory types, such as sensory sensation seeking.

However, items on the AASP include questions about other factors that may influence our sensory experiences. For example, there are items about visual attention (for example, ‘I miss the street, building, or room signs when trying to go somewhere new’), and affective reactions towards sensory stimuli (for example, ‘I become frustrated when trying to find something in a crowded drawer*’*). As such, the AASP, while producing clear group differences, measures a broader set of perceptual processes and affective responses and not basic sensory function. Thus, there is a need for a more fine-grained questionnaire to dissect each perceptual process with greater precision.

More recently the Sensory Over-responsivity Scale (SensOR) was developed to assess sensory processing disorder (SPD), or more narrowly sensory over-responsivity [6]. Sensory Over-Responsivity (SOR) is defined as an exaggerated response to one or more types of sensory stimuli [6]. The SensOR measures SOR by asking how many sensations are experienced as aversive (for example, labels in clothes). The SensOR was developed in combination with an examiner-administered assessment of response to real-world stimuli; these two measures correlate moderately (r = .47) [6]. This association suggests that the SensOR is a valid and reliable tool to investigate sensory issues. However, like the Sensory Profile, items on the SensOR include affective reactions towards sensory stimuli (for example, participants have to rate which items in the environment bother them), so again, it is not measuring basic sensory sensitivity.

There is thus a need for a basic sensory perception questionnaire that does not assess social and affective aspects. In contrast to the SensOR and the Adult Sensory Profile, the Sensory Perception Quotient (SPQ), reported here for the first time, only investigates basic sensory sensitivity, with no reference to affective response (see Table 1). The SPQ was developed to quantify individual differences in sensory perception in the general population and adults with ASC, based on the assumption that this trait shows variance following a normal distribution in community samples. All five of the main sensory modalities (vision, hearing, touch, smell and taste) were included.

**Table 1:** Examples of differences between the adult sensory profile (ASP), the sensory over-responsivity scale (SensOR) and the sensory perception quotient (SPQ)

**SPQ Item**	**ASP item**	**SensOR item**	**Difference**
I would be able to tell when an elevator/lift started moving.	I avoid escalators and/or elevators because I dislike the movement.	These aspects related to movement bother me - going up or down escalators	SPQ items aim to measure basic perception (for example, detection).
			AASP and SensOR items also include behavioural and affective responses towards sensations.
If I look at a pile of blue sweaters in a shop that are meant to be identical, I would be able to see differences between them.	I like to go to places that have bright lights and that are colourful.	These visual sensations bother me: - brightly coloured or patterned materials (for example, clothes, drapes, wallpaper)	

The purpose behind development of the SPQ is to assist researchers studying sensory perception in adults. Additionally, the SPQ is intended to be useful for occupational therapists and other clinicians. In summary, the objectives of the current study were: (1) to explore the factor structure of the new SPQ, its reliability and its concurrent validity with a previously validated instrument; (2) to investigate if adults with and without ASC show differences on the SPQ; and (3) to explore if sensory sensitivity is correlated with autistic traits, both across and within groups.

## Methods

The Cambridge University Psychology Research Ethics Committee approved the study.

### Participants

Adults with ASC were recruited via an online volunteer database hosted by the Autism Research Centre, University of Cambridge. These volunteers were invited to take part in the online questionnaire study via the website at http://www.autismresearchcentre.com. Data from participants with no ASC diagnosis were collected via a parallel website at http://www.cambridgepsychology.com. Only participants who reported to have no psychiatric history were included in the control group.

All participants first filled in background information including age, sex, and history of psychiatric conditions including when, where and by whom they were diagnosed. Only participants with an ASC diagnosis made by a qualified professional (psychologist or psychiatrist) were included in the ASC group. To validate diagnoses in the ASC group and to screen control participants, we used the Autism Spectrum Quotient (AQ) [22]. We used a standard inclusion criterion of an AQ cut-off score of 26 and above for the ASC group, and a score below 26 for the control group [22,23]. Nine participants with ASC and 43 control participants were excluded on the basis of the AQ cut-off score, leaving 359 participants: n = 196 participants with ASC (100 males, 96 females) and n = 163 control participants (49 males, 114 females). Participants were not reimbursed for taking part in the current study.

### Autism Spectrum Quotient

All participants completed the adult version of the AQ. The AQ is a short, 50-item questionnaire measuring autistic traits, with five subscales (social skills, attention switching, attention to detail, imagination and communication) [22,23]. A score of 0 is assigned to the responses ‘definitely agree’ and ‘slightly agree’ and a score of 1 for ‘slightly disagree’ and ‘definitely disagree’ for half the items, and the reverse for the other half, designed to avoid a response bias. Total scores could therefore range from 0 to 50, with higher scores indicating more autistic traits. Results from the AQ have been replicated cross culturally [24,25] and across different ages [26]. The AQ shows also good test-retest reliability [22-24].

### Raven’s Progressive Matrices Test

IQ was estimated using a short online adaptation of the Raven’s Progressive Matrices (60 items) as a timed performance task [27]. The Raven’s Progressive Matrices Test has the advantage of being language-free and it can be used for a wide range of ages and cross-culturally (Raven, 2000). Each item consisted of a pattern consisting of a missing section, and the participant is asked to select the option that accurately completes the pattern shown (with 15 seconds allowed for each item).

### Sensory over-responsivity scale

The SensOR Scale consists of a self-report inventory that measures over-responsivity in several sensory domains (touch, vision, hearing, smell, taste, and proprioception) [6]. Participants indicated which items in their daily environment bother them (for example, in the tactile domain: labels in clothing*,* or in the auditory domain: a clock ticking*)*. The SensOR went through item analysis and reduction prior to item selection for this edition. The current SensOR edition consists of 76 items (28 tactile items, 20 auditory items, 9 taste items, 9 movement items, 5 vision and 5 smell items). The internal consistency reliability for the total test is high (r = .97) and concurrent validity of the SensOR score with the Sensory Profile score for sensory reactivity and sensory avoiding is moderate (r = .50) [6].

### Sensory Perception Quotient (SPQ): Instrument development

We first generated items for vision, hearing, touch, smell and taste. Next, these items were given to experts in ASC and participants with and without ASC for feedback on wording and applicability. Words with affective aspects (such as like/dislike) were avoided. Instead we focused on basic detection and/or discrimination abilities (for example, ‘I would be able to detect if a strawberry was ripe or not by smell alone*’*). In addition we checked that where possible, items were worded appropriately given that adults with ASC prefer very specific, clear, unambiguous wording (for example, ‘I would notice if someone added 5 grains of salt to my water*’*). Half the items were worded to identify hypersensitive (that is, low thresholds) and half were worded to identify hyposensitive items (that is, high thresholds), to avoid bias (see Table 2 for a complete item breakdown). When scoring, hyposensitive items were reversed, so that a low total SPQ score indicates more reactivity.

**Table 2:** Categories and subcategories of the sensory perception quotient (SPQ)

**Main categories**				
Touch	Hearing	Vision	Smell	Taste
(20)^a^	(20)	(20)	(16)	(16)
**Subcategories**				
Pressure (5)	Amplitude (5)	Acuity (5)	Social (4)	Salty (4)
Temperature (5)	Frequency (5)	Brightness (5)	Danger (4)	Sweet (4)
Pain (5)	Vestibular (5)	Colour (5)	Food (4)	Sour (4)
Vibration (5)	Complexity (5)	Motion (5)	Neutral (4)	Bitter (4)

^a^In brackets is the number of items per category.

The final version of the SPQ covered items for vision, hearing, touch, smell and taste. We aimed to investigate basic sensory processing and therefore included main receptors for each modality and/or the characteristics of the environment relevant to each sense (see Table 2). We developed questions about receptors from different modalities and corresponding environmental stimuli (for example, Pacinian corpuscles are tactile receptors, which are sensitive to vibrations). The only exception was olfaction since there are too many receptors to develop items for each one [28]. Instead, the main functions of olfaction were included. Equal weightings were assigned to vision, hearing and touch, on the basis that none of the senses is more important than others. However we included slightly fewer items for taste and smell since humans do not tend to depend on chemical senses as much as other animals [28]. Humans are microsmatic, having a poor sense of smell, whereas many animals are macrosmatic, having a good sense of smell [28]. Finally, we included a larger number of items in total, so as to be able to reduce these after an item analysis.

### Scoring

Participants were asked to indicate to what extent they agreed or disagreed with each statement on a Likert scale (0 = strongly agree, 1 = agree, 2 = disagree, and 3 = strongly disagree). All item responses were summed [29], with a lower score indicating higher sensory sensitivity. Advantages of this scale are that it avoids uncertain answers, and it can be used for multidimensional constructs.

### Procedure

Participants could complete the Raven’s Progressive Matrices, AQ, SensOR and SPQ tasks online in their preferred order, and were allowed to log out between tests.

## Results

### Descriptive statistics

PASW Statistics 18 was used to analyse the data. Tests of normality (Kolmogorov-Smirnov test; KS) showed that that SPQ scores were normally distributed (*P* > .20). There was no significant difference between the groups on age or IQ (*P* > .05). However as expected, the ASC group had a higher mean AQ score than the control group (t = 42.95, *P* < .0001) (see Table 3).

**Table 3:** Descriptive characteristics of the Autism Spectrum Condition (ASC) and control groups

	**ASC group**	**Control group**	**Group difference**
N	196	163	-
Age in years (SD)	38.7 (12.7)	36.8 (12.3)	No, *P* > .05
AQ score (range from 0 to 50)	40.4 (5.1)	15.4 (6.2)	Yes, *P* < .0001
Raven score (range from 0 to 60)	50.3 (10.5)	51.5 (7.4)	No, *P* > .05

AQ, Autism Spectrum Quotient.

### Principal component analysis

To investigate the underlying factor structure of the SPQ a principal component analysis (PCA) was conducted, using the Varimax rotation method. The PCA was first run for the data from the control participants, and subsequently repeated in the ASC group. Extraction of underlying dimensions was based on inspection of the scree plots, which suggested that the SPQ is composed of two underlying dimensions in both the control group and the ASC group.

Next, factor loadings were visually inspected. For the control group, most items (38 in total) loaded on Factor 1, whilst only a few items loaded on Factor 2. Items were retained if they showed a high factor loading (≥.35) on the one factor and a low loading (≤.35) on the other. We excluded ambiguous items that showed high loadings ≥ .35 on both factors [30]. A separate PCA for the ASC group showed similar results. In this group, 43 items loaded highly on Factor 1. In addition, 35 items that loaded on Factor 1 in the control group also loaded on Factor 1 in the ASC group (see Table 4). For Factor 2, the loadings across both groups were low and inconsistent. These results suggest that one factor, encompassing 35 items, consistently assesses sensory reactivity traits in both adults with ASC and controls.

**Table 4:** Item loadings for factors 1 (Fac1) and 2 (Fac2) for all 92 items of the Sensory Perception Quotient (SPQ), and item breakdown of the SPQ domains and subdomains (for example, taste-salty)

**Factor loadings and item break down**		**Factor loading**				**Item domain**
**Item**		**Control group**		**ASC group**		
		Fac1	Fac2	Fac1	Fac2	
1	I would notice if someone added 5 grains of salt to my cup of water.	.34	-.18	.38	-.35	Taste-salty
2^a^	I would be able to distinguish different people by their smell.	.60	.23	.42	-.22	Smell-social
3	I wouldn’t notice if someone added a spoonful of sugar to my tea.	.02	.15	.35	.40	*Taste-sweet*
4	I wouldn’t be afraid of hurting myself when falling off my bike at high speed.	.04	-.01	.03	.52	*Touch-pain*
5	I wouldn’t be able to detect the motion of the blades of a rotating fan even when it is at minimum speed.	.09	.36	.30	.48	*Vision-motion*
6	The sound of a piano and a violin playing the same note seems very similar to me.	-.02	.22	.26	.39	Hearing-complexity
7^a^	I would be able to detect if a strawberry was ripe by smell alone.	.38	.25	.55	-.11	Smell-food
8	I would be able to distinguish milk chocolate and dark chocolate by their taste alone.	.31	.33	.47	.09	Taste-sweet
9	I cannot tolerate hot showers (above 40°C /105°F).	.16	-.28	.23	-.12	Touch-pain
10	I wouldn’t need an anaesthetic to cope with a dental procedure, such as a cavity-filling.	-.02	-.03	.13	.39	*Touch-pain*
11	I would have to wait for 10 minutes for a hot drink to cool down before swallowing it, otherwise it would be too hot for me.	.27	-.19	.26	-.16	Touch-temperature
12^a^	I would be able to visually detect the change in brightness of a light each time a dimmer control moved one notch.	.49	.04	.60	-.23	Vision-brightness
13	I wouldn’t be able to detect large objects, such as parked cars, clearly on a dark night.	-.12	.46	.33	.42	*Vision-brightness*
14^a^	I would notice if someone added 5 drops of lemon juice to my cup of water.	.48	.13	.56	-.08	Taste-sour
15	I would be the last person to detect if something was burning.	.43	.30	.58	.46	*Smell-danger*
16	I wouldn’t be able to feel the vibrations from loud music if I was sitting next to the loud speaker (for example, at a concert).	.04	.19	.38	.65	*Touch-vibration*
17	I wouldn’t be able to feel a small volume change in music as a difference in vibration on my skin.	-.30	.19	-.63	.02	*Touch-vibration*
18	I can’t hear the TV when it is quiet, even when other people can.	.01	.41	.33	.21	*Hearing-loudness*
19^a^	I would be able to hear a leaf move if blown by the wind on a quiet street.	.43	.05	.59	-.20	Hearing-loudness
20	I wouldn’t be able to taste the difference between two pieces of dark chocolate.	.34	.18	.60	.10	*Taste-sweet*
21^a^	I would be able to taste the difference between two brands of salty potato chips/crisps.	.48	.17	.60	-.11	Taste-salty
22	When people are talking the words seem to merge together.	-.32	.60	-.08	.42	*Hearing-complexity*
23	I can only look at bright colours for a brief period of time.	.34	-.61	.48	-.47	Vision-colour
24	I would lose my balance very easily if I was standing on one foot with my eyes closed.	.25	-.51	.03	-.41	Hearing-vestibular
25	I wouldn’t be able to smell a barbecue from 60 feet (20 metres) away.	.40	.27	.52	.41	*Smell-food*
26	I can’t spin round and round without falling over.	-.17	.42	-.24	.33	*Hearing-vestibular*
27	I wouldn’t notice a 10 degree difference in temperature of the weather.	.22	.36	.24	.57	*Touch-temperature*
28	I can drink tea/coffee ‘straight’, without needing to add milk or sugar.	.20	-.26	.23	.06	*Taste-bitter*
29	I can’t hear the bass in music.	.30	.35	.45	.42	*Hearing-frequency*
30	I would be able to smell the difference between freshly cut grass and uncut grass.	.27	.14	.49	-.05	Smell-neutral
31^a^	I wouldn’t be able to feel the label at the back of my shirt even if I thought about it.	.50	-.29	.57	.21	*Touch-pressure*
32^a^	I can hear electricity humming in the walls.	.43	-.36	.57	.44	Hearing-frequency
33^a^	I notice the flickering of a desktop computer even when it is working properly.	.52	-.32	.61	-.46	Vision-motion
34	I wouldn’t be able to tell if milk is off simply by smelling it.	.22	.41	.52	.38	*Smell-food*
35^a^	I would be able to notice a tiny change (for example, 1 degree) in the temperature of the weather.	.46	.09	.58	-.32	Touch-temperature
36^a^	I would be able to feel a one millimetre cut in my skin.	.36	.09	.44	-.13	Touch-pain
37	I would be able to see the individual blades in a rotating fan even if it was at maximum speed.	.29	-.20	.26	-.44	Vision-motion
38^a^	I would be able to tell the weight difference between two different coin sizes on the palm of my hand, if my eyes were closed.	.42	.03	.62	-.25	Touch-pressure
39	I wouldn’t get dizzy on a carousel/merry-go-round, even at high speed.	.03	.07	-.02	.24	*Hearing-vestibular*
40	I can’t see written words on a page that other people can see.	-.12	.37	-.14	.63	*Vision-acuity*
41	I would be able to distinguish between two oranges purely by their taste.	.48	.22	.31	-.11	Taste-sour
42^a^	I couldn’t distinguish a familiar person and a stranger by their smell.	.41	.25	.42	.11	*Smell-social*
43^a^	I couldn’t detect if bread is stale purely by its smell.	.38	.06	.58	.16	*Smell-food*
44	I can’t tell if my clothes are clean or dirty by smell alone.	.30	.35	.43	.48	*Smell-neutral*
45^a^	I would be able to detect the sound of a vacuum cleaner from any room in a two-storey building.	.44	.04	.51	-.03	Hearing-frequency
46	I wouldn’t notice the difference between even and uneven ground when driving over it sitting in the back seat of a car.	.38	.31	.46	.47	*Touch-vibration*
47	I would be able to drink a cup of boiling water straight after it had been poured from the kettle.	-.03	.04	.16	.49	*Touch-pain*
48	I couldn’t tell two types of green apples apart purely from their colour.	.31	.26	.49	.36	*Vision-colour*
49	I would be able to distinguish between an old and a new book by their smell.	.25	.18	.58	-.06	Smell-neutral
50	I would be able to read a street sign from a distance of 100 feet (30 metres).	-.09	.29	.22	-.01	Vision-acuity
51	I can’t tell if cars passing me on the street are going at different speeds.	.15	.35	.25	.51	*Vision-motion*
52	I would be able to notice if someone added 5 grains of sugar to my glass of water.	.37	-.05	.35	-.30	Taste-sweet
53	I would have difficulty seeing a single leaf clearly even on a tree that is close up.	.21	.36	.34	.56	Vision-acuitity
54	I wouldn’t taste if someone added a whole teaspoon of salt to my glass of water.	.20	.37	.23	.56	*Taste-salty*
55^a^	I would be able to feel the elastic holding up my socks if I stop and thought about it.	.57	-.11	.57	-.11	Touch-pressure
56	I can’t taste the difference between ripe and non-ripe fruit.	.21	.41	.39	.56	*Taste-sweet*
57	I would be able to stand on one foot for fifteen seconds without wobbling.	.15	-.44	.22	-.06	*Hearing-vestibular*
58^a^	I would be able to taste the difference between apparently identical pieces of candy.	.37	-.27	.59	-.27	Taste-sweet
59^a^	I notice the weight and pressure of a hat on my head.	.49	-.06	.61	-.19	Touch-pressure
60^a^	I would feel if a single hair touched the back of my hand.	.59	-.08	.64	-.20	Touch-pressure
61^a^	If I was walking along, I would be able to feel a passing truck’s vibrations even if my eyes were closed.	.54	-.00	.69	-.07	Touch-vibration
62^a^	I would be able to smell the smallest gas leak from anywhere in the house.	.64	-.09	.71	-.29	Smell-danger
63^a^	I wouldn’t notice if someone changed their perfume, by smell alone.	.43	.19	.59	.23	*Smell-social*
64	I would be able to tell when an elevator/lift started moving.	.25	.07	.53	.10	Hearing-vestibular
65	I can hear dog whistles very easily in the park.	.32	-.15	.50	-.30	Hearing-frequency
66	I wouldn’t taste the difference between different types of lettuce leaves.	.45	.27	.17	.36	*Taste-bitter*
67	I couldn’t taste if there were two slices of lemon in my glass of water if I was drinking it with my eyes closed.	.31	.19	.48	.51	*Taste-sour*
68^a^	I can’t go out in bright sunlight without sunglasses.	.47	-.26	.55	-.35	Vision-brightness
69	I would be able to read small print, such as a serial number on the back of a DVD, at 10 feet (3 metres) away.	-.17	.02	.04	-.38	Vision-acuity
70	I get motion sickness easily (for example, car sickness or sea sickness)	.21	-.01	-.01	-.11	Vision-motion
71^a^	I would be able to feel a change in the temperature of a cup of coffee after it had sat for 1 minute.	.51	.07	.56	-.12	Touch-temperature
72	I can’t hear very low frequency sounds, such as low voices.	.09	.52	.44	.27	*Hearing-frequency*
73^a^	I would be the first to hear if there was a fly in the room.	.61	-.08	.70	-.27	Hearing-loudness
74^a^	If I look at a pile of blue sweaters in a shop that are meant to be identical, I would be able to see differences between them.	.51	-.26	.68	-.25	Vision-colour
75^a^	I wouldn’t detect a new smell in my house instantly before anyone else.	.55	.26	.58	.19	*Smell-neutral*
76	I have perfect pitch: for example, I could repeat a musical tone without any cue.	-.42	.04	-.21	.16	Hearing-complex
77	I would be able to bite into a lemon without any problems.	.15	-.32	.18	.25	*Taste-sour*
78	I wouldn’t need to wear a coat in the winter, even when it is zero degrees outside.	-.01	-.03	-.12	-.52	*Touch-temperature*
79	I wouldn’t be able to match the colour of a sweater in the shop with the colour of my trousers at home.	.22	.07	.14	.34	*Vision-colour*
80	I wouldn’t hear every single note when listening to music.	.39	-.01	.33	.21	*Hearing-frequency*
81^a^	I would be able to smell the difference between most men and women.	.51	.25	.59	-.19	Smell-social
82	I choose to wear muted colours.	.14	-.33	.41	-.31	Vision-brightness
83	I listen to music at minimum loudness.	.15	-.22	.31	-.27	Hearing-loudness
84^a^	I would be able to hear each note in a chord even if there were 10 notes.	.38	-.13	.43	-.25	Hearing-complexity
85^a^	I close curtains to avoid bright lights.	.46	-.45	.49	-.37	Vision-acuity
86	I wouldn’t be able to hear differences in sound if the same instrument played the same note at different times.	.27	-.12	.50	.20	*Hearing-complex*
87^a^	I would be able to distinguish two brands of coffee by their smell, even with my eyes closed.	.50	.01	.66	-.16	Smell-food
88^a^	I can see dust particles in the air in most environments.	.50	-.37	.63	-.42	Vision-acuity
89^a^	I wouldn’t be able to taste the difference between two brands of tomato sauce if they had different concentrations of salt.	.43	.26	.60	.35	*Taste-salty*
90^a^	I would be able to smell the smallest amount of burning from anywhere in the house.	.62	-.10	.76	-.20	Smell-danger
91^a^	If my mobile phone was vibrating in my pocket I would be quick to sense it.	.43	-.01	.59	.12	Touch-vibration
92	I find it difficult to see individual stars on a clear night.	.15	.21	.43	.47	*Vision-acuitity*

Sensory domains included: vision, hearing, touch, smell and taste. Italic items are linked to high thresholds (hyposensitive). Non-italic items are linked to lower thresholds (hypersensitive).

^a^ Items included in the short version of the SPQ (those items with factor loadings > .35 in one factor for both groups and a low loading (≤.35) on the other factor).

### Item distribution analysis

In addition, an item distribution analysis was conducted. Items on which more than 70% of the participants gave the same response were excluded, since items with very little variance are not informative [31]. Four such items were identified: item, 8, 16, 47 and 54. On these items most participants ‘strongly agreed’ (scoring 0). None of these items loaded on Factor 1.

### Item reduction

In sum, the PCAs suggest that one factor including 35 items consistently assesses traits related to sensory reactivity in both a control group and a clinical group (see Table 4). All these 35 items show considerable response variation. Out of the 35 items loading onto Factor 1, 10 items assessed reactivity to touch, 10 items assessed smell, 6 vision, 5 hearing, and 4 taste. Most of the items (31 out of 35) were hypersensitive items.

### Reliability

For all 92 items, the split-half reliability was high (Spearman-Brown = .91, *P* < .0001). Additionally, Cronbach’s alpha suggested excellent internal consistency for both the full 92 item version of the SPQ (α = .92) and for the reduced 35-item version (α = .93).

### Concurrent validity

**Figure 1:**
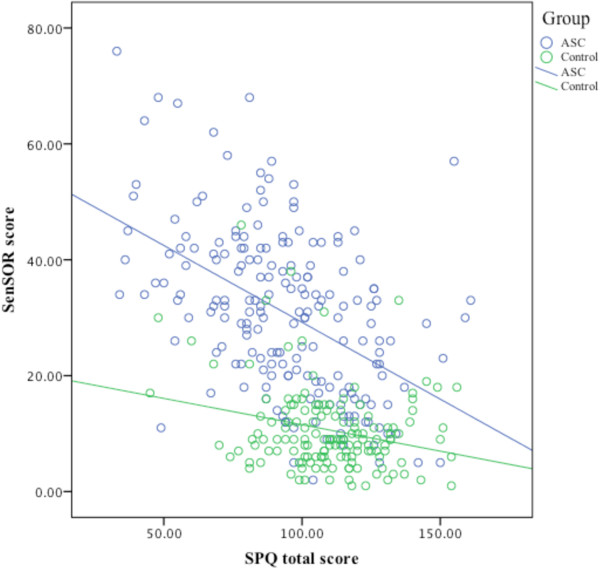
**The correlation between the Sensory Perception Quotient (SPQ) with the sensory over-responsivity (SensOR).** The lower the score on the SPQ the more sensitive a person is, and the higher the score on the SensOR the more sensitive. ASC, autism spectrum conditions.

To test the concurrent validity of the SPQ the association with the SensOR was examined. High scores on the SensOR and low scores on the SPQ represent more reactivity to stimuli in the environment. The total SPQ and SensOR correlated moderately (r = -.50, *P* < .0001) both across groups and within the ASC (r = -.49, *P* = .007) and control group (r = -.23, *P* = .004) (see Figure 1). The concurrent validity was lower for the item reduced version of the SPQ (r = -.20, *P* = .0001).

### Differences between the groups

A MANOVA with group and sex as fixed factors showed that groups differed significantly on the SPQ total scores (F(6,339) = 13.44, *P* < .0001) (see Table 5). Post hoc tests showed that groups differed significantly on the total SPQ, the item-reduced version, and for all subscales other than smell. Additionally, sex differences were found for the total SPQ (F (1) = 4.71, *P* < .005), and for smell and taste reactivity; females in both groups had lower scores on the SPQ (more sensitive) (see Table 5). Gender had however no effect on SPQ-short scores. Results from the SenSOR are reported elsewhere [32].

**Table 5:** The mean sensory perception quotient (SPQ) scores before and after item reduction and for all subscales for vision, hearing, touch, smell and taste, for females and males with and without Autism Spectrum Condition (ASC)

	**Sex**	**SPQ**	**SPQ**	**SPQ**	**SPQ**	**SPQ**	**SPQ Smell**	**SPQ Taste**
		**Full**	**Short**	**Vision**	**Hearing**	**Touch**		
**ASC group**	Both	92.95	38.55	22.12	22.56	18.35	14.56	14.34
		(±26.61)	(18.68)	(±6.61)	(±5.41)	(±6.29)	(±8.34)	(±6.39)
	**Male**	97.5	40.70	22.77	23.37	23.37	15.93	15.75
		(±25.24)	(19.84)	(±6.36)	(±6.36)	(±5.33)	(±8.29)	(±5.78)
	**Female**	88.21	36.25	21.54	21.54	18.58	13.12	12.87
		(±27.30)	(±17.20)	(±6.84)	(±6.84)	(±5.39)	(±8.23)	(±6.69)
**Control group**	**Both**	108.96	43.01	27.12	25.85	22.74	14.85	16.25
		(±20.53)	(±14.67)	(±5.35)	(±4.79)	(±5.20)	(±5.85)	(±5.09)
	**Male**	110.06	43.01	27.25	25.58	22.70	16.00	17.85
		(±17.53)	(±14.67)	(±4.16)	(±4.57)	(±4.46)	(±6.10)	(±5.50)
	**Female**	106.84	44.57	27.09	25.95	22.74	14.34	15.53
		(±21.71)	(±14.60)	(±5.82)	(±4.93)	(±5.52)	(±5.69)	(±4.75)
**Group difference?**		6.71^a^	8.20^a^	58.33^a^	26.80^a^	41.74^a^	.89	16.80^a^
F (p)		(.01)	(004)	(.0001)	(.0001)	(.0001)	(.34)	(.000)
**Sex difference?**		8.42^a^	2.41	2.85	2.18	.26	6.73^a^	21.12^a^
F (p)		(.004)	(.12)	(.09)	(.14)	(.60)	(.01)	(.0001)

Standard deviations are shown in brackets. The SPQ full includes all 92 items and SPQ short includes the 35 item reduced version. In addition, group and sex differences are presented.

^a^*P* values ≤ .01

### Correlation between Sensory Perception Quotient and autistic traits

The total SPQ was correlated with the AQ across groups (r = -.38, *P* = .0001) and within the ASC group (r = -.18, *P* = .009), and marginally within the control group (r = -.15, *P* = .06). A higher score on the AQ corresponds with more autistic traits, while lower scores on the SPQ suggest a lower sensory threshold and thus a higher sensory sensitivity. The reduced SPQ also correlated with the AQ (r = -.14, *P* = .007). There was no correlation between SPQ total scores and age (*P* = .58) or IQ (*P* = .95).

## Discussion

The current study reports the development and validation of the Sensory Perception Quotient (SPQ), a new sensory questionnaire that provides a quantitative measurement of individual differences in basic sensory perception. The SPQ shows excellent internal consistency and good validity. Adults with ASC reported being more sensitive than control participants to sensory stimuli in vision, hearing, touch and taste, but not smell. Reliable sensory sensitivity measures for adults with ASC are needed since sensory symptoms are now recognized as being at the core of ASC. Past questionnaire-based studies already highlighted the importance of sensory reactivity in ASC, but often used parent reports [5,8-16]. Since it is easier to judge your own sensory experiences the SPQ is an important new self-report questionnaire. This new questionnaire also adds to a battery of new dimensional measures (the Autism Spectrum Quotient (AQ), the Empathy Quotient (EQ) and the Systemizing Quotient (SQ)) that seek to provide a metric of the spectrum on which ASC lies, and the relationship between ASC and variation in the general population [22,33,34].

With regards to item analysis, principal component analyses suggest that one factor including 35 items consistently assesses traits related to sensory sensitivity, in both the clinical group and the control group. Both the short 35-item version and the total 92-item version of the SPQ showed high internal consistency. In addition the SPQ correlated with the AQ, meaning greater sensory reactivity is associated with more autistic traits. Furthermore the SPQ was moderately correlated to a validated sensory scale, the SensOR. It is not surprising that the SPQ and SensOR are not perfectly related, as the SensOR also assesses the affective response to sensory stimuli, whilst the SPQ focuses on basic sensory perception only. Future studies need to test whether SPQ scores correlate to laboratory-measured reactivity measures. In addition, future studies are needed to investigate other test characteristics, such as test-retest reliability.

A limitation of this study is the uneven sex ratio between the groups (more females in the control group). However the ASC and control group differed on the SPQ even when sex was included as a factor. Sex had an effect on the full SPQ, in line with findings from the Sensory Profile [35]. Females in both groups have lower scores on the SPQ, meaning women report being more sensitive. Since females with ASC reported they were more sensitive, the question arises if sensory issues affect females with ASC to a greater extent. A recent study shows that women with ASC report more life-time sensory issues compared to men with ASC [36]. This is important and needs more research since most studies neglect female participants with ASC and mostly include males. Finally, given that this is the first report of the SPQ, future studies are necessary to generate a normative dataset.

## Conclusions

The SPQ is a reliable and valid new tool to measure sensory sensitivity in adults with and without ASC. Adults with ASC report more sensory sensitivity than controls, and this may have important implications for how they manage their everyday lives. The SPQ is a useful standardized measure for basic sensory perception in adults with ASC, other clinical disorder and neurotypical adults. Uses of the SPQ include assisting researchers studying sensory issues in adults (for example, in phenotyping studies), and to assist clinicians, such as occupational therapists, in assessing the sensory needs of people with autism.

## Abbreviations

AASP: adolescent/adult sensory profile; ASC: autism spectrum conditions; ASP: adult sensory profile; SenSOR: sensory over-responsivity scale; KS: Kolmogorov-Smirnov test; PCA: principal component analysis; SPQ: sensory perception quotient.

## Competing interests

The authors of this paper report that they have no biomedical financial interests or potential conflicts of interest.

## Authors’ contributions

TT and SBC designed the SPQ. TT collected the data and TT and RH carried out the data analyses. All authors were involved in writing the manuscript and approved the final version.
